# Bariatric surgery and its impact on depressive symptoms, cognition, brain and inflammation

**DOI:** 10.3389/fendo.2023.1171244

**Published:** 2023-07-06

**Authors:** Lenka Kotackova, Radek Marecek, Andrei Mouraviev, Ariana Tang, Milan Brazdil, Michal Cierny, Tomas Paus, Zdenka Pausova, Klara Mareckova

**Affiliations:** ^1^ Brain and Mind Research, Central European Institute of Technology, Masaryk University (CEITEC MU), Brno, Czechia; ^2^ Department of Neurology, St. Anne’s University Hospital and Faculty of Medicine, Masaryk University, Brno, Czechia; ^3^ Departments of Psychiatry and Neuroscience, Faculty of Medicine and Centre Hospitalier Universitaire Sainte-Justine, University of Montreal, Montreal, QC, Canada; ^4^ Hospital for Sick Children, University of Toronto, Toronto, ON, Canada; ^5^ Departments of Physiology and Nutritional Sciences, University of Toronto, Toronto, ON, Canada; ^6^ Bariatric Clinic, Breclav Hospital, Breclav, Czechia

**Keywords:** bariatric surgery, obesity, visceral fat, cortical thickness, depression, cognition, inflammation, longitudinal

## Abstract

**Background:**

Obesity has been associated with depressive symptoms and impaired cognition, but the mechanisms underlying these relationships are not well understood. It is also not clear whether reducing adiposity reverses these behavioral outcomes. The current study tested the impact of bariatric surgery on depressive symptoms, cognition, and the brain; using a mediation model, we also examined whether the relationship between changes in adiposity after the surgery and those in regional thickness of the cerebral cortex are mediated by changes in low-grade inflammation (as indexed by C-reactive protein; CRP).

**Methods:**

A total of 18 bariatric patients completed 3 visits, including one baseline before the surgery and two post-surgery measurements acquired at 6- and 12-months post-surgery. Each visit consisted of a collection of fasting blood sample, magnetic resonance imaging of the brain and abdomen, and assessment of depressive symptoms and cognition.

**Results:**

After surgery, we observed reductions of both visceral fat (p< 0.001) and subcutaneous fat (p< 0.001), less depressive symptoms (p< 0.001), improved verbal reasoning (p< 0.001), and reduced CRP (p< 0.001). Mediation analyses revealed that the relationships between the surgery-related changes in visceral fat and cortical thickness in depression-related regions are mediated by changes in CRP (ab=-.027, SE=.012, 95% CI [-.054, -,006]).

**Conclusion:**

These findings suggest that some of the beneficial effects of bariatric surgery on brain function and structure are due to a reduction of adiposity-related low-grade systemic inflammation.

## 1 Introduction

We are in the midst of a global epidemic of obesity and diabetes. World Health Organization estimates that – since 1980 - the number of adults living with obesity has doubled, and the number of adults living with diabetes has almost quadrupled (1). Obesity is associated not only with adverse cardiometabolic health outcomes, but also with impaired cognition (2, 3) and depressive symptoms (4). The relationship between obesity and impaired cognition has been described in the context of inhibitory control and working memory (5, 6), verbal memory, and processing speed (7) Moreover, mid-life obesity has been reported as a risk factor for cognitive impairment and dementia in later life (8–10).

Bariatric surgery is the most effective treatment of both obesity and obesity-related diseases, including type 2 diabetes mellitus. Various mechanisms have been proposed to explain its effectiveness, including mechanical restriction and lower food intake, action of neuropeptides (incretin, GLP-1) (11), malabsorption, changes in bile-acid circulation (12, 13) or satiety regulation (14). It was also suggested that bariatric surgery might improve the pre-operative cognitive deficits, and that the improved self-esteem might have positive effects on mood (15).

Previous research reported associations between obesity and brain structure (16, 17). A meta-analysis of 21 studies on obesity and voxel-based morphometry demonstrated consistent associations between obesity-related variables and lower gray-matter volume in several regions of the cerebral cortex and in the cerebellum (18). In an independent dataset, higher body mass index (BMI) was associated with lower volumes of regions implicated in executive control (18). In the UK Biobank dataset, higher total body fat was associated with lower volumes of gray matter in subcortical nuclei, and variations in structural properties of white matter (19) Two previous studies investigated possible consequences of bariatric surgery on regional volumes (20) and regional thickness (21) of the cerebral cortex, with inconsistent results, possibly due to small sample sizes. While several mechanisms explaining the effects of bariatric surgery on the brain and behavior have been proposed (22, 23), a better understanding of the relationships is needed.

C-reactive protein (CRP) is a simple, relatively inexpensive, and widely available blood test used to detect inflammation, including subclinical inflammation (24). Several studies have shown a causal association between (visceral) adiposity and low-grade inflammation (24–26), including studies in experimental animals (27). CRP, a marker of both acute and chronic (low-grade) inflammation (28, 29) were reported in overweight and obese adults (30). It has been also suggested that inflammatory markers including the CRP might change after bariatric surgery, but the literature is inconsistent (31). While some studies demonstrated an increase in inflammatory markers in the early postoperative period in response to tissue healing (32, 33), a meta-analysis (34) showed a consistent decrease in inflammatory markers CRP and IL-6 observed six and more months after bariatric surgery.

The current study investigated the impact of bariatric surgery on depressive symptoms and cognition. We also aimed to determine the specific impact of post-surgery changes in adiposity, as indexed by BMI, visceral and subcutaneous fat, on post-surgery changes in these behavioral outcomes. We hypothesized that bariatric surgery-related reduction of BMI, visceral and subcutaneous fat will predict less inflammation, less depressive symptoms, better cognition and more cortical thickness in the relevant brain regions. Finally, we also hypothesized that the relationships between the post-surgery changes in adiposity and the changes in cortical thickness of brain regions associated with the behavioral outcomes will be mediated by changes in CRP. To test these hypotheses, we used a longitudinal design, with a baseline measurement before the surgery and two measurements afterward (at 6- and 12-months post-surgery).

## 2 Materials and methods

### 2.1 Participants

This report includes a total of 18 participants with severe obesity (3 men, 15 women; mean age 48.6 ± 10.6 years; mean BMI 43.3 ± 6.7 kg/m²) who underwent bariatric surgery. All participants completed three visits, the baseline visit 2 weeks before the surgery, as well as two follow-up visits at 6 months and 12 months after the surgery (see Procedures). All participants were recruited during their pre-surgery examination, received complete information about the study, and passed the following exclusion criteria: presence of neurological or psychiatric disorders, medication other than for diabetes and hypertension, previous gastric, esophageal, brain or bariatric surgery, gastro-intestinal inflammatory disease, severe food allergy pregnancy and any contraindications of magnetic resonance (MR) imaging. The presence of a psychiatric disorder was assessed during a psychological interview but symptoms of depression or binge-eating disorder were not included under the exclusion criteria. We approached a total of 33 potential participants; 8 participants were excluded based on the above exclusion criteria and 7 eligible participants declined to participate, thus leaving a final sample of 18 participants. Further demographic information, the presence of co-morbidities such as diabetes or hypertension, and blood levels of basic cardiometabolic disease (CMD) indicators are provided in **Table 1A**. The study was approved by the Research Ethics Committee at Masaryk University, and all participants provided written informed consent.

Table 1Adiposity, cardiometabolic disease risk factors, and neuropsychological test performance before and after the bariatric surgery.

**Table:** 

A – Adiposity and cardiometabolic disease risk factors before and after the bariatric surgery																								
		Visit 1 (2 weeks before bariatric surgery)						Visit 2 (½ years after bariatric surgery)						Visit 3 (1 year after bariatric surgery)						Pairwise comparison				
		n=18						n=18						n=18						1st vs. 2nd visit		2nd vs. 3rd visit		1st vs. 3rd visit
		Mean		SD		Range		Mean		SD		Range		Mean		SD		Range		*p*		*p*		*p*
Age		48.67		10.57		33 - 69		48.97		10.57		33 - 69		49.67		10.68		34 - 70						
Sex		M: 3						M: 3						M: 3										
		W: 15						W: 15						W: 15										
Weight		127.78		22.85		98 - 172		103.72		17.03		71-132		96.67		17.69		65-124		***		***		***
BMI (kg/m²)		43.33		6.67		35.11 – 63.95		35.22		5.08		26.08 – 49.08		32.7		4.68		24.24 – 43.87		***		**		***
Visceral fat (cm^3^)		50.5		23.47		23.6 – 109.5		34.37		20.04		8.05 – 76.76		30.53		19.56		9.48 – 75.73		***		*		***
Subcutaneous fat (cm^3^)		177.80		33.72		107.8 – 219.9		126.95		42.42		75.83 – 198.6		123.83		48.28		68,.4 – 198.1		***		NS		***
Systolic blood pressure (mmHg)		138.33		14.75		110 - 160		125		10.43		100 - 140		126.39		10.26		110 - 145		**		NS		***
Diastolic blood pressure (mmHg)		86.33		8.58		80 - 110		82.33		6.15		70 - 98		82.22		4.28		80 - 90		NS		NS		NS
Hypertension		Yes: 11												Yes:3										
		No:7												No: 15										
Type 2 diabetes mellitus		Yes: 3												Yes:1										
		No: 15												No: 17										
Glucose (mmol/l)		5.68		1.58		4.1 – 9.6		4.88		0.82		3.9 - 7		5.02		1.28		3.9 – 9.6		**		NS		NS
Insulin (mmol/l)		25.69		18.48		38 – 70.76		14.44		11		4.83 – 53.05		11.13		5.24		5.11 – 22.67		*		NS		***
Total cholesterol levels (mmol/l)		5.34		1.18		2.88 – 8.13		5.34		1.05		3.93 – 7.74		5.13		0.86		3.78 – 6.66		NS		NS		NS
Triglycerides (mmol/l)		1.61		0.69		0.87 – 3.02		1.4		0.69		0.65 – 2.81		1.4		0.61		0.66 – 2.92		NS		NS		NS
HDL-cholesterol (mmol/l)		1.22		0.27		0.84 – 1.74		1.3		0.32		0.83 - 2		1.4		0.3		0.87 – 1.96		NS		NS		*
LDL-cholesterol (mmol/l)		3.46		1.07		0.93 – 5.54		3.41		0.85		1.93 – 5.27		3.1		0.71		1.82 – 4.39		NS		NS		NS
NonHDL-cholesterol (mmol/l)		4.1		1.09		2.04 – 6.9		4.05		0.93		2.87 – 6.54		3.74		0.73		2.56 – 5.04		NS		NS		NS
CRP (mg/l)		9.04		7.25		1.3 - 26		5.29		4.58		0.3 – 18.6		3.88		3.84		0.3 – 13.5		*		**		***

B - Neuropsychological test performance before and after the bariatric surgery																								
	Visit 1 (2 weeks before bariatric surgery)						Visit 2 (½ year after bariatric surgery)						Visit 3 (1 year after bariatric surgery)						Pairwise comparison					
	n=18						n=18						n=18						1^st^ vs. 2^nd^ visit		2^nd^ vs. 3^rd^ visit		1^st^ vs. 3^rd^ visit	
	Mean		SD		Range		Mean		SD		Range		Mean		SD		Range		*p*		*p*		*p*	
Assessment of mood																								
BDI	14.05		10.77		1.0 – 39.0		6.27		7.56		0 - 27		6.27		7.56		0 - 27		***		NS		***	
Executive functions and attention																								
Digit span total																								
Subtest A	9.22		2.67		3 - 14		9.11		2.19		3 - 13		9.83		2.79		3 - 14		NS		NS		NS	
Subtest B	9.22		3.62		3 - 16		9.28		2.74		4 - 15		9.06		2.88		5 - 15		NS		NS		NS	
Verbal reasoning test (Perf)	70.69		11.62		52.5 – 97.5		78.47		12.99		57.5 - 100		74.86		15.06		47.5 - 100		*		NS		NS	
Stroop test (Perf)	98.84		1.34		95.8 - 100		98.84		1.04		96.7 - 100		98.89		1.18		96.7 - 100		NS		NS		NS	
Flag attention test (PoE)	0.06		0.11		0 – 4.5		0.16		0.56		0 – 2.4		0.27		0.9		0 – 3.85		NS		NS		NS	
Trail making test																								
Subtest A	66.41		22.01		38.13 – 108.51		61.86		25.67		32.25 – 130.39		61.13		20.45		35.96 – 108.96		NS		NS		NS	
Subtest B	100.11		41.18		50.42 – 213.8		103.87		68.47		51.9 – 344.9		87.78		41.02		42.11 – 210.37		NS		NS		NS	
CT in depression-related ROIs	2.63		0.07		2.5 – 2.74		2.63		0.08		2.49 – 2.75		2.61		0.1		2.43 – 2.75		NS		NS		NS	
CT in verbal reasoning-related ROIs	2.5		0.12		2.23 – 2.82		2.51		0.14		2.21 – 2.86		2.47		0.15		2.2 – 2.85		NS		*		NS	

Adiposity and cardiometabolic disease risk factors are described in 1A, neuropsychological test performance in 1B.

HDL, high density lipoproteins; LDL, low density lipoproteins; CRP, C-reactive protein; *p < 0.05, **p < 0.01, ***p < 0.001, NS, non-significant.

BDI, Beck depression inventory; Perf, Performance; PoE, percentage of errors; CT, cortical thickness.

*p < 0.05, **p < 0.01, ***p < 0.001, NS, non-significant.

### 2.2 Procedures

Each of the 18 participants completed all three visits. Each visit included collection of a fasting blood sample, assessments of depressive symptoms and cognition, and MRI of the brain and abdomen.

#### 2.2.1 Blood

Blood samples collected after overnight fasting were used to analyze levels of CMD indicators, namely glucose, insulin, triglycerides, total cholesterol, high-density lipoprotein (HDL)-cholesterol, low-density lipoprotein (LDL)-cholesterol, non-high-density lipoprotein (non-HDL)-cholesterol, and C-reactive protein (CRP).

#### 2.2.2 Depressive symptoms and cognition

Depressive symptoms were assessed using Beck Depression Inventory (BDI) (35). The assessment of cognition included Trail making test (TMT) (36), Digit span (37)), Verbal reasoning test (38) Stroop test (39), and Flag attention test (40, 41). The TMT was used as an indicator of visual scanning, visuomotor speed, and executive function (36). It consisted of two parts; during the first part, participants were asked to connect numbers, during the second part, participants were asked to connect numbers with letters. We used the delta trace score (TMT part B minus part A) as a measure of set shift (42). During the Digit span, which tests verbal short-term memory (37), the participants were asked to memorize several digits and repeat them in the same (subtest A) or reversed (subtest B) order. The verbal reasoning test (38) was used to test verbal abilities; performance is indexed by the number of correct and incorrect answers, as well as by the average response time. The Stroop test (39) was used to test the ability to resist cognitive interference; performance is indexed by the success rate of the whole test (average number of patterns correctly found for the whole test converted to a percentage) and the average time to solve the tests. Finally, the Flag attention test (40, 41) allowed us to quantify selective attention and speed of perception by the degree of accuracy during stimuli evaluation, and the number of stimuli processed in a given time.

#### 2.2.3 MR imaging: acquisition

All participants underwent structural MR imaging of the brain and abdomen at 3T Siemens Prisma MRI scanner located at the Central European Institute of Technology, Masaryk University (CEITEC MU) during each visit. The MR protocol included a T1-weighted MPRAGE scan (TR=2400ms; TE=2.12ms; TI=1060ms; FA=8deg; FOV=230x230mm; matrix size=288x288; 256 sagittal slices with slice thickness=0.8mm; voxel size=0.8mm isotropic; acceleration factor: GRAPPA, PE=2; the image comprised entire head) and DIXON fat-only Controlled Aliasing In Parallel Imaging (CAIPI; TR=4ms; TE1 = 1.27ms; TE2 = 2.5ms; FA=9deg; FOV=499x390mm; matrix size=320x250; 72 transversal slices with slice thickness=3mm; voxel size=1.56x1.56x3mm; acceleration factor: GRAPPA, PE=2; slice partial Fourier 7/8; all images comprised L3 to L5 part of spine).

### 2.3 MR imaging: analysis

Cortical thickness was estimated using FreeSurfer 7.1.1. First, we ran default recon-all command for all participants and visits individually. Next, we created an unbiased individual-specific template from all three visits for each participant with recon-all and –base/-tp directives. Finally, each visit was registered to an individual’s template by recon-all and –long directive. Subsequently, we extracted overall cortical thickness in a total of 30 depression-related regions as identified by (42), namely right (R) middorsolateral frontal cortex, left (L) frontopolar cortex (lateral), R ventromedial prefrontal cortex, R frontal medial cortex, L middorsolateral frontal cortex, R and L superior frontal sulcus, R frontal precentral sulcus, R and L temporal pole, R superior temporal gyrus, R and L superior temporal sulcus (anterior), R and L superior temporal sulcus (posterior), R and L supramarginal/angular gyrus, R and L fusiform gyrus, R lingual gyrus, R and L precuneus, L parieto- occipital sulcus, R and L anterior cingulate sulcus (rostral), L and R insula (anterior), R and L anterior cingulate sulcus (caudal), R parahippocampal gyrus, as well as overall cortical thickness in a total of 6 verbal reasoning-related regions as identified by Chen et al. (2017), namely R inferior frontal gyrus/insula, dorsal anterior cingulate cortex, supplementary motor area, L and R angular gyrus, and R temporo-parietal junction. The binary images that defined the regions of interest (ROIs) were mapped to the Freesurfer’s FSaverage template with mri_vol2surf command. Then, the participants’ estimates of whole-brain cortical thickness were registered to FSaverage using mri_surf2surf command, and the average cortical thickness for each ROI was computed using mri_segstats command. The outputs from all steps were visually inspected for any segmentation and spatial registration errors. Cortical thickness in all the depression-related regions was averaged to get a single value of cortical thickness in depression-related regions. Similarly, cortical thickness in all the verbal reasoning-related regions was averaged to get a single value of cortical thickness in verbal reasoning-related regions.

Visceral and subcutaneous fat were quantified manually using Slice-O-Matic 5.0 (Tomovision, Quebec, Canada). This segmentation process was performed in a blinded manner, with the abdominal scans anonymized. First, CAIPI scans were converted from NIFTI into DICOM format using the software Mango (University of Texas, Texas, USA). Then, the DICOM scans were segmented using Slice-O-Matic 5.0. Scans at the L4 intervertebral disc level were then used to quantify visceral and subcutaneous fat volumes (cm^3^). Adipose tissue within the abdominal cavity and outside of organ compartments were marked as visceral fat; tissue outside of the abdominal muscles and spinal erectors was marked as subcutaneous fat.

### 2.4 Statistical analyses

All statistical analyses were carried out using JMP version 10.0.0 (SAS Institute Inc., Cary, NC). First, we log‐transformed variables that did not follow normal distribution, and then we used repeated-measures ANOVA to examine the impact of bariatric surgery on adiposity (BMI, visceral and subcutaneous fat in the L4 region), depressive symptoms (BDI), cognition (Trail making test, Verbal reasoning test, Digit span, Stroop test, Flag test), and cortical thickness in the regions implicated in depression and cognitive functioning. Effect size for these analyses is reported with η^2^. Since most of the bariatric surgery-related effects showed between Visit 1 and Visit 2 (6 months after the surgery) but not between Visit 3 and Visit 2, the next set of analyses focused on the changes between Visit 2 and Visit 1. Linear regression assessed the relationships between post-surgery changes (Visit 2 – Visit 1) in adiposity and post-surgery changes (Visit 2 – Visit 1) in CRP, depressive symptoms, cognitive functioning, and cortical thickness in the regions implicated in depression and cognitive functioning. Finally, we aimed to determine whether changes in CRP (an index of inflammation, during Visit 2 – Visit 1) might mediate the relationship between changes in adiposity (Visit 2 – Visit 1) and changes in cortical thickness (Visit 2 – Visit 1) in the regions implicated in depression and cognitive functioning. The mediation analyses were done using the PROCESS macro for SPSS (IBM SPSS Statistics, IBM Corp, Armonk, NY). Conditional indirect effects were assessed for significance using bootstrapped bias corrected 95% confidence intervals constructed around indirect effect estimates, based on 10 000 bootstrapping iterations.

## 3 Results

### 3.1 Effects of bariatric surgery on body adiposity

As expected, repeated measures ANOVA showed lowering effects of bariatric surgery on all measures of body adiposity: BMI (Wilks’ Lambda = 0.089, F (2,16) = 81.91, p = 3.9x10^-9^, η^2 = ^0.91), weight (Wilks’ Lambda = 0.112, F (2,16) = 63.66, p = 2.4x10 ^-8^, η^2 = ^0.89), visceral fat (Wilks’ Lambda = 0.160, F (2,13) = 34.12, p = 6.7x10^-6^, η^2 = ^0.84), and subcutaneous fat (Wilks’ Lambda = 0.254, F (2,13) = 19.12, p = 1.3x10^-4^, η^2 = ^0.75). These results remained significant also when controlling the model for sex: BMI (Wilks’s Lambda = 0.23, F(2,15)=24.81, p<0.001, η^2 = ^0.77), weight (Wilks’s Lambda = 0.35, F(2,15)=9.32, p<0.001, η^2 = ^0.55), visceral fat (Wilks’s Lambda = 0.236, F(2,12)=31.54, p<0.001, η^2 = ^0.62), subcutaneous fat (Wilks’s Lambda = 0.242, F(2,12)=18.77, p=0.002, η^2 = ^0.46). Subsequent *post-hoc* comparisons between visits indicated lower BMI, weight, and visceral fat after the surgery (after 6 months vs. pre-surgery baseline), as well as between the first and second post-surgery measurements. For subcutaneous fat, these differences were found between the baseline and the first/second post-surgery measurements but not between first and second post-surgery measurements See statistics in **Table 1A**.

### 3.2 Effects of bariatric surgery on cardiometabolic disease risk factors

Also as expected, repeated measures ANOVA showed lowering effects of bariatric surgery on systolic blood pressure (Wilks’ Lambda = 0.368, F(2,16) = 13.75, p = 3.3x10^-4^, η^2^ = .63), insulin (Wilks’ Lambda = 0.403, F(2,16) = 11.86, p = 0.001, η^2 = ^0.60), and CRP (Wilks’ Lambda = 0.231, F(2,16) = 26.58, p =8.2x10^-6^, η^2 = ^0.73), and increasing effects of bariatric surgery on HDL-cholesterol (Wilks’ Lambda = 0.563, F(2,16) = 6.22, p = 0.010, η^2 = ^0.44). There was only a trend for the effect of bariatric surgery on glucose (Wilks’ Lambda = 0.535, F(2,16) = 6.95, p = 0.007). These results did not change significantly even when controlling for sex: systolic blood pressure (Wilks’ Lambda = 0.328, F(2,15) = 15.33, p < 0.001, η^2 = ^0.57), insulin (Wilks’ Lambda = 0.202, F(2,15) = 8.16, p = 0.010, η^2 = ^0.37), CRP (Wilks’ Lambda = 0.581, F(2,15) = 5.42, p =0.017, η^2 = ^0.42), HDL-cholesterol (Wilks’ Lambda = 0.552, F(2,15) = 6.01, p = 0.012, η^2 = ^0.40), and glucose (Wilks’ Lambda = 0.527, F(2,15) = 6.7, p = 0.008). Follow-up *post hoc* comparisons between visits indicated that, after (vs. before) bariatric surgery, there was lower systolic blood pressure, glucose, insulin, CRP and higher HDL-cholesterol; most of these differences were found between the baseline and the first or second post-surgery measurement but not between the first and second post-surgery measurements. There was no significant effect of bariatric surgery on diastolic blood pressure, triglycerides, and total, LDL- and non-HDL-cholesterol. See statistics in **Table 1A**.

### 3.3 Effects of bariatric surgery on depressive symptoms and cognitive functioning

Repeated measures ANOVA showed effects of bariatric surgery on BDI (Wilks’ Lambda = 0.277, F(2,16) = 20.88, p =3.5x10^-5^, η^2 = ^0.72) and Verbal reasoning (Wilks’ Lambda = 0.542, F(2,16) = 6.76, p = 0.007, η^2 = ^0.46). These results remained significant when controlling the model for sex: BDI (Wilks’s Lambda = 0.57, F(2,15)=5.58, p=0.015, η^2 = ^0.43), Verbal reasoning (Wilks’ Lambda = 0.54, F(2,15) = 6.34, p = 0.010, η^2 = ^0.31). *Post-hoc* comparisons between visits indicated that there were fewer depressive symptoms and better verbal-reasoning skills after the surgery; again, these differences were found between the baseline and the first/second post-surgery measurement but not between the first and second post-surgery measurements. There was no significant effect of bariatric surgery on Digit span, Trail making test, Stroop test, or the Flag attention test. See statistics in **Table 1B**.

### 3.4 Effects of bariatric surgery on cortical thickness in depression-related and verbal reasoning-related ROIs

Repeated measures ANOVA did not show an effect of bariatric surgery on cortical thickness in depression-related regions (Wilks’ Lambda = 0.689, F(2,16) = 3.61, p = 0.51, η^2 = ^0.31) but showed an effect on cortical thickness in verbal reasoning-related regions (Wilks’ Lambda = 0.546, F(2,16) = 6.66, p = 0.008, η^2 = ^0.45). See statistics in **Table 1B**. When controlling for sex, these results remained not significant in depression-related regions (Wilks’ Lambda = 0.981, F(2,15) = 0.15, p = 0.863, η^2 = ^0.02) but also not significant in verbal reasoning-related regions (Wilks’ Lambda = 0.869, F(2,15) = 1.13, p = 0.349, η^2 = ^0.13).

Given the fact that most of the measurements of adiposity, CMD-risk factors, depressive symptoms, and cognition showed changes between Visit 1 and 2 (before the surgery vs. 6 months after the surgery; see **Table 1**) but not between Visit 2 and 3 (6 vs. 12 months after the surgery), the subsequent analyses focused on the changes between Visit 1 and 2.

### 3.5 Post-surgery changes in adiposity and CRP

**Figure 1:**
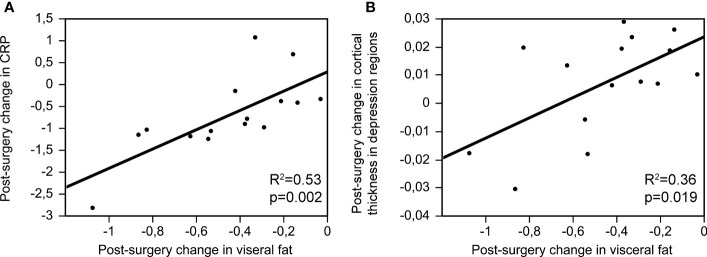
Post-surgery changes in visceral fat (Visit 2 – Visit 1) and their relationship with post-surgery changes in CRP **(A)**; beta=0.72, p=0.002, R^2 = ^0.53) and cortical thickness in depression-related regions **(B)**; beta=0.59, p=0.019, R^2 = ^0.36).

While post-surgery changes (Visit 2 – Visit 1) in BMI and subcutaneus fat did not show a relationship with post-surgery changes in CRP (BMI: beta=0.21, p=0.396; subcutaneous fat: beta=0.05, p=0.855), there was a strong and significant relationship between post-surgery changes in visceral fat and CRP (beta=0.72, p=0.002, R^2 = ^0.53; **Figure 1A**). These results remained significant when controlling for sex: post-surgery changes (Visit 2 – Visit 1) in BMI and subcutaneous fat did not show a relationship with post-surgery changes in CRP (BMI: beta=0.23, p=0.372; subcutaneous fat: beta=0.17, p=0.581), but there was a strong and significant relationship between post-surgery changes in visceral fat and CRP (beta=0.76, p=0.001, R^2 = ^0.62).

### 3.6 Post-surgery changes in adiposity and depression and cortical thickness in depression-related regions

There was no relationship between post-surgery changes in adiposity (BMI, visceral fat or subcutaneous fat) and post-surgery changes in depressive symptoms (BMI: beta=-0.44, p=0.067; visceral fat: beta=-0.09, p=0.749; subcutaneous fat: beta=0.05, p=0.865). While post-surgery changes in BMI and subcutaneous fat did not show a relationship with cortical thickness in depression-related regions (BMI: beta=0.33, p=0.183; subcutaneous fat: beta=0.38, p=0.159), there was a strong and significant relationship between post-surgery changes in visceral fat and cortical thickness in depression-related regions (beta=0.59, p=0.019, R^2 = ^0.36; **Figure 1B**).

These results remained significant when controlling for sex: post-surgery changes in adiposity (BMI, visceral fat or subcutaneous fat) and post-surgery changes in depressive symptoms (BMI: beta=-0.46, p=0.055; visceral fat: beta=-0.13, p=0.659; subcutaneous fat: beta=-0.07, p=0.810). While post-surgery changes in BMI and subcutaneous fat did not show a relationship with cortical thickness in depression-related regions (BMI: beta=0.33, p=0.193; subcutaneous fat: beta=0.42, p=0.180), there was a strong and significant relationship between post-surgery changes in visceral fat and cortical thickness in depression-related regions (beta=0.59, p=0.026, R^2 = ^0.36).

### 3.7 Post-surgery changes in adiposity and verbal reasoning and cortical thickness in verbal reasoning-related regions

There was no relationship between adiposity (BMI, visceral or subcutaneous fat) and verbal reasoning (BMI: beta=-0.21, p=0.398; visceral fat: beta=-0.09, p=0.762; subcutaneous fat: beta=0.16, p=0.569) or cortical thickness in verbal reasoning regions (BMI: beta=-0.37, p=0.132; visceral fat: beta=-0.32, p=0.251; subcutaneous fat: beta=0.17; p=0.553). These results remained not significant for verbal reasoning (BMI: beta=-0.21, p=0.427; visceral fat: beta=-0.09, p=0.772; subcutaneous fat: beta=0.20, p=0.541) or cortical thickness in verbal reasoning regions (BMI: beta=-0.35, p=0.157; visceral fat: beta=-0.29, p=0.302; subcutaneous fat: beta=0.32; p=0.301) even when controlling for sex.

### 3.8 Do post-surgery changes in CRP mediate the relationship between post-surgery changes in visceral fat and cortical thickness in the depression regions?

**Figure 2:**
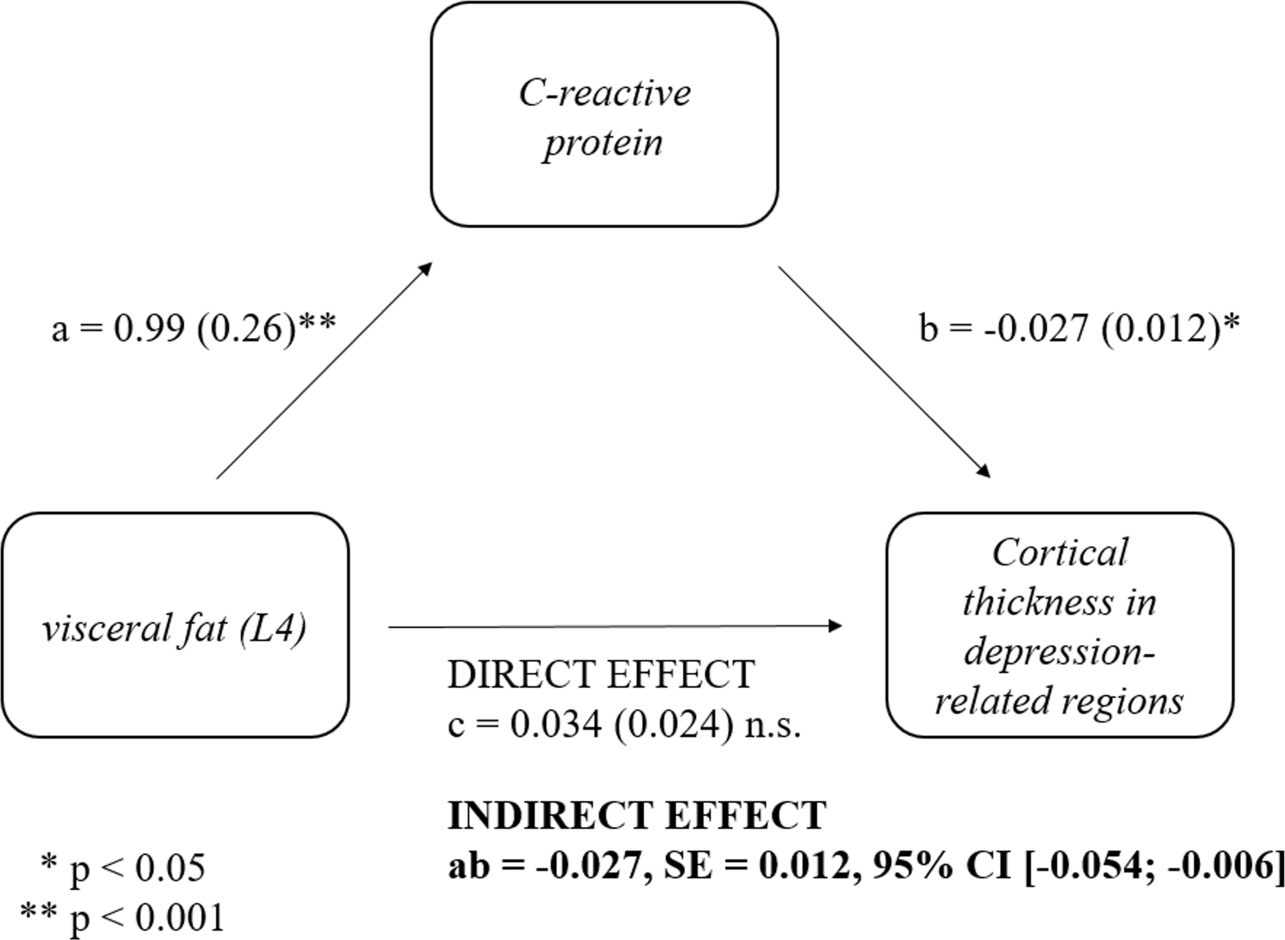
Mediatory role of post-surgery changes in C-reactive protein (CRP) in the relationship between post-surgery changes in visceral fat and post-surgery changes in cortical thickness in depression regions. Post-surgery changes in CRP (Visit 2 – Visit 1) mediated the relationship between post-surgery changes in visceral fat (ab=-.027, SE=.012, 95% CI [-.054, -,006]) and post-surgery changes in cortical thickness in depression regions. Standardized coefficients are provided.

The mediation analysis showed that the relationship between the post-surgery changes in visceral fat and post-surgery changes in cortical thickness in the depression-related regions was completely mediated by post-surgery changes in CRP (ab=-.027, SE=.012, 95% CI [-.054, -,006]; see **Figure 2**). These results remained significant (ab=-.028, SE=.012, 95% CI [-.056, -.007]) even when controlling for sex.

## 4 Discussion

This study examined the effect of bariatric surgery on depressive symptoms, cognition, and the brain, and tested whether the bariatric surgery-associated changes in low-grade inflammation might mediate the relationships between adiposity and cortical thickness in depression- andverbal-reasoning-relatedd regions. We demonstrated that bariatric surgery was associated with a reduction of depressive symptoms and improved verbal reasoning, changes that paralleled those in post-surgical reduction of both visceral and subcutaneous fat, and CRP. The post-surgical decrease in CRP mediated the relationship between post-surgery reduction of adiposity (visceral fat) and post-surgery increase of cortical thickness in depression-related cortical regions. This is consistent with our previous research, which showed that visceral fat is associated with circulating markers of systemic inflammation more closely than subcutaneous fat (43). Moreover, these findings suggest that the bariatric surgery-related change in systemic inflammation is the underlying mechanism explaining the relationship between reduced adiposity and improved mental health.

We did not observe similar relationships between post-surgery change in visceral fat and post-surgery change in cortical thickness in verbal reasoning-related regions, suggesting that the impact of bariatric surgery on depressive symptoms and verbal reasoning are driven by different mechanisms. While reduced adiposity and inflammation seem to underlie the effect of bariatric surgery on the reduction of depressive symptoms, the mechanisms underlying the relationship between bariatric surgery and improved verbal reasoning are less clear and might include greater confidence and more frequent participation in socially and mentally stimulating activities after the weight reduction (44) or novel mechanisms such as a reduction in the adipokine-induced inflammatory response (45). It is also important to point out that there were no effects of bariatric surgery on other cognitive skills including attention, working memory or ability to resist cognitive interference, and that the improved reaction time on verbal reasoning test might be simply associated with participants familiarity with the test they have already done at visit 1. Therefore, the impact of bariatric surgery on verbal reasoning should be interpreted with caution.

The current study has several strengths including the longitudinal design, MRI of both the brain and abdomen, as well as the combination of MRI with rich behavioral assessments. Still, there are also several limitations. First, given our relatively small sample size, we were powered to detect large and medium effects but not small effects. Future research should replicate our findings using a larger sample. Second, we did not include a control group as the aim of our study was to test the effect of bariatric surgery on depressive symptoms, cognition and the brain rather than to compare participants with and without obesity or an obese control group treated by other methods or untreated. Further, the self-reporting nature of the survey might have biased results regarding depressive symptomatology. Future studies could also examine the duration of obesity as an important factor for cognitive outcomes. Any duration of obesity might result in adverse consequences for the brain, but it is more likely that a longer duration of obesity or obesity at a critical period of brain development will have greater consequences. Future research might also consider the influence of other factors, such as changes in non-adipose tissue, exercise habits (46), sleep quality (47), gastrointestinal hormone secretion (48, 49) and the impact of estrogens and a woman’s menstrual cycle (50, 51) on brain structure and function after bariatric surgery (53).

## Data availability statement

The raw data supporting the conclusions of this article will be made available by the authors, without undue reservation.

## Ethics statement

The study was approved by the Research Ethics Committee at Masaryk University, and all participants provided written informed consent.

## Author contributions

LK - investigation, formal analysis, writing original draft, and visualization, RM – software and data curation, AM – methodology and software, AT – software and formal analysis, MB – resources and funding acquisition, MC – resources, TP – conceptualization and methodology, reviewing and editing the manuscript, ZP – conceptualization and funding acquisition, reviewing and editing the manuscript, KM – supervision, formal analysis, reviewing and editing the manuscript. All authors contributed to the article and approved the submitted version.
